# Stimulating Fracture Healing in Ischemic Environments: Does Oxygen Direct Stem Cell Fate during Fracture Healing?

**DOI:** 10.3389/fcell.2017.00045

**Published:** 2017-05-04

**Authors:** Katherine R. Miclau, Sloane A. Brazina, Chelsea S. Bahney, Kurt D. Hankenson, Thomas K. Hunt, Ralph S. Marcucio, Theodore Miclau

**Affiliations:** ^1^Department of Orthopaedic Surgery, University of CaliforniaSan Francisco, CA, USA; ^2^Zuckerberg San Francisco General Hospital, Orthopaedic Trauma InstituteSan Francisco, CA, USA; ^3^Harvard CollegeCambridge, MA, USA; ^4^Department of Small Animal Clinical Science and Department of Physiology, Michigan State UniversityEast Lansing, MI, USA; ^5^Department of Orthopaedic Surgery, University of PennsylvaniaPhiladelphia, PA, USA; ^6^Department of Surgery, University of CaliforniaSan Francisco, CA, USA

**Keywords:** fractures, bone, repair, ischemia, oxygen, stem cell, differentiation, stimulation

## Abstract

Bone fractures represent an enormous societal and economic burden as one of the most prevalent causes of disability worldwide. Each year, nearly 15 million people are affected by fractures in the United States alone. Data indicate that the blood supply is critical for fracture healing; as data indicate that concomitant bone and vascular injury are major risk factors for non-union. However, the various role(s) that the vasculature plays remains speculative. Fracture stabilization dictates stem cell fate choices during repair. In stabilized fractures stem cells differentiate directly into osteoblasts and heal the injury by intramembranous ossification. In contrast, in non-stable fractures stem cells differentiate into chondrocytes and the bone heals through endochondral ossification, where a cartilage template transforms into bone as the chondrocytes transform into osteoblasts. One suggested role of the vasculature has been to participate in the stem cell fate decisions due to delivery of oxygen. In stable fractures, the blood vessels are thought to remain intact and promote osteogenesis, while in non-stable fractures, continual disruption of the vasculature creates hypoxia that favors formation of cartilage, which is avascular. However, recent data suggests that non-stable fractures are more vascularized than stable fractures, that oxygen does not appear associated with differentiation of stem cells into chondrocytes and osteoblasts, that cartilage is not hypoxic, and that oxygen, not sustained hypoxia, is required for angiogenesis. These unexpected results, which contrast other published studies, are indicative of the need to better understand the complex, spatio-temporal regulation of vascularization and oxygenation in fracture healing. This work has also revealed that oxygen, along with the promotion of angiogenesis, may be novel adjuvants that can stimulate healing in select patient populations.

## Introduction

Ischemia, the restriction of blood supply to tissues, leads to hypoxic and nutrient-deficient environments and results in decreased cellular metabolism and proper tissue functioning, including reduced fracture repair (Lu et al., 2007; Miedel et al., 2013). Although many of the mechanisms regarding ischemia's inhibitory effect on wound healing remain unknown, preclinical and clinical experiments have been conducted to analyze the role of vasculature in fracture healing. The literature overwhelmingly supports the critical role of blood supply in the complex process of skeletal regeneration.

Musculoskeletal disorders—any injury or disorder that affects the muscles, bones, and joints —are the second leading cause of disability worldwide with the fourth greatest impact on overall health (Woolf and Pfleger, 2003). Within the United States alone, musculoskeletal disorders currently affect half of all adults and 75% of people over 65. With each passing year, these conditions only become more of a burden as the population ages (Weinstein et al., 2014). In particular, as the lifespan of the average American increases with improvements in healthcare, nutrition, and living conditions, rates of osteoporosis and osteoarthritis in the elderly are increasing rapidly, leading to a dramatic rise in the number of geriatric fractures. Each year, fractures affect 15 of every 1,000 people worldwide and occur at rates of 15 million fractures per year in the US. Of these 15 million fractures, an estimated 10–15% will not heal properly (Einhorn, 1995).

Fracture treatment is associated with an enormous societal burden secondary to direct and indirect recovery costs. In 2010, trauma surpassed cardiovascular disease as the leading healthcare cost burden in the US, accruing $21 billion a year in trauma-related visits to medical facilities and accounting for 6.6% of the total cost of hospital care in the US (Allison Russo et al., 2004). Costs of these direct treatments represent only 20% of the economic burden of trauma-related injuries. The remaining 80% is due to the indirect costs of productivity loss, as approximately half of all individuals suffering fractures do not return to work within the first 6 months of recovery (Kanakaris and Giannoudis, 2007).

Impaired healing exacerbates the economic burden of fracture-related conditions, particularly in instances of non-union. In the US alone, nearly 100,000 non-union cases—fractures characterized by failure to heal within 9 months post-injury, and lack of progress toward union, as demonstrated by radiogram, within 3 months—are treated each year, with each patient accruing an estimated $11,333 in direct and indirect costs (Dickson et al., 1995). In 1994, a report demonstrated that ~$14.6 million is spent annually to treat delayed union—failure to achieve union by 6 months post-injury—and non-union fractures (Kanakaris and Giannoudis, 2007). This colossal economic burden imposed on the United States health care system motivates our need to better understand the various factors responsible for impaired fracture healing and to develop more effective treatments.

Evidence suggests that compromised vascularity is a leading cause of non-union conditions, making the treatment of ischemic fractures a viable target to reduce US healthcare spending. Approximately 46% of fracture patients with accompanying vascular injuries experience impaired bone healing, which is significantly higher than the average 10% non-union rate (Dickson et al., 1995). In cases with vascular-related comorbidities such as diabetic angiopathy and trauma-related extensive soft tissue damage, blood flow is often compromised at the site of the fracture, thereby impeding the comprehensive vascular response necessary for proper bone regeneration and highlighting ischemia as a primary risk factor.

## Clinical relevance

Although the effect of ischemia on fracture healing and the mechanism of bone regeneration under hypoxic conditions has been studied in murine models, this work has yet to be translated to *in vivo* human studies. Few clinical studies have addressed the role of ischemia in fracture repair. Dickson et al. retrospectively evaluated the prognosis of healing as a function of arterial injury in tibial fractures and found that open tibia fracture patients presenting with arterial occlusion have a significantly higher rate of delayed union and non-union (Dickson et al., 1994). Avany et al. demonstrated a 50% incidence of arterial occlusion among tibial non-union patients (Arany et al., 1980), and Dietz et al. reported that tibial non-unions in chronically ischemic limbs healed after the arterial supply was restored to normal (Deitz et al., 1989). These three clinical studies illustrate a clear correlation between a lack of blood supply to injured tissue and impaired fracture healing as a result. Impaired healing of diabetic foot ulcers serves as another example of the importance of vascularization and oxygenation in wound healing. Diabetic foot ulcers occur in 15% of all diabetic patients and are accompanied by prolonged hypoxia and inadequate angiogenesis. Decreased wound vascularization associated with diabetic foot ulcers is associated with impaired mobilization and homing of endothelial progenitor cells (EPCs), and a decrease in VEGF levels (reviewed in: Guo and Dipietro, 2010). Studies demonstrate that the mobilization of EPCs to diabetic foot ulcers in patients is reversible through treatment with moderate hyperoxia, which suggests that ischemia induced by prolonged hypoxia contributes to compromised healing. (Liu and Velazquez, 2008)

Clinical analyses of healing rates for patients with impaired vascularization supports the importance of adequate blood supply during fracture healing. Within a population free of chronic disease, the risk of non-union ranges from 10 to 15% (Einhorn, 1995). This figure is increased to nearly 50% when fractures are accompanied by impaired vasculature (Dickson et al., 1994, 1995). Several non-traumatic risk factors are associated with vasculature disease that may also contribute to an increased incidence of delayed union and non-union (Buza and Einhorn, 2016). Diabetics experience approximately two to three times longer fracture healing than non-diabetic patients (Mehta et al., 2010), and diabetes is associated with decreased angiogenesis (Abaci et al., 1999; Galiano et al., 2004). A higher proportion of fractures in smokers result in non-union or delayed union compared to non-smokers (Castillo et al., 2005); one study has shown that tibial healing times were 62% longer in smokers compared to non-smokers (Schmitz et al., 1999). Cigarette smoking has long been associated with cardiovascular disease (Benowitz and Burbank, 2016) and defective angiogenesis (Ejaz and Lim, 2005), but nicotine has also been shown to have angiogenic activities (Heeschen et al., 2001). No definitive studies examining the effect of smoking or nicotine on angiogenesis after fracture have been performed to date. Elderly patients heal more slowly than young adult patients (Green et al., 2005), and research has shown that angiogenesis is delayed during fracture healing in older animals (Lu et al., 2008a). As evident by these findings, many patient populations are at risk for compromised fracture healing.

## Role of vasculature during fracture healing

While clinical studies clearly demonstrate the necessity of the vasculature for efficient healing, the specific role(s) that the vasculature plays is unknown. Long bone fractures are almost always accompanied by vascular disruption in the surrounding soft tissues, creating a hematoma around the fracture site. Interestingly, the blood vessels that couple angiogenesis and osteogenesis appear to be unique (Kusumbe et al., 2014). The vasculature serves at least two important functions during fracture repair: delivery of nutrients to the damaged tissue, and transport of cells to the healing fracture site. Blood vessels provide oxygen and nutrients to the site of injury that are necessary for cell survival. Additionally, the blood supply is also a route for inflammatory cells and other cell types that are recruited from systemic sources to the fracture site. Vasculature may also provide important signals that help regulate the process of bone fracture repair (Bahney et al., 2015; Hu et al., 2017).

## Effect of oxygen levels on stem cell differentiation during fracture healing

Mechanical stability is associated with the mode of healing at the fracture site (Carter and Giori, 1991; Claes and Heigele, 1999). Rigidly stabilized fractures heal through intramembranous ossification (Willenegger et al., 1971; Le et al., 2001), a process in which stem cells located in the periosteum and endosteum differentiate directly into osteoblasts that form bone directly (Thompson et al., 2002; Colnot, 2008). In contrast, fractures that are not rigidly stabilized heal primarily through endochondral ossification with a small amount of direct bone formation in the periosteum and endosteum (Probst and Spiegel, 1997; Hankemeier et al., 2001). During endochondral ossification stem cells differentiate into chondrocytes, which then transform into osteoblasts to form the new bone. Clinically, most fractures in humans heal through a combination of intramembranous and endochondral ossification (Urist and Johnson, 1943; Hak et al., 2014).

The differences in vascularity between bone and cartilage may provide some insight into these two different mechanisms of fracture healing: bone is a highly vascularized tissue, while cartilage is completely devoid of blood vessels. Therefore, one possibility is that in a stabilized environment the blood supply is more intact than in a non-stable environment, and this favors direct bone formation. In contrast, in non-stable environments disruption to the blood supply may favor chondrogenesis due to localized hypoxia (Probst and Spiegel, 1997; Malda et al., 2003), which leads to a large cartilage callus that eventually transforms to bone as the vasculature invades. This idea is supported by observations that chondrogenesis of articular chondrocytes proceeds *in vitro* much more efficiently in cultures that have reduced oxygen levels (Murphy and Polak, 2004), suggesting that oxygen levels may play a role in differentiation of chondrocytes and osteoblasts.

While the data on the effects of oxygen on skeletogenic stem cells is still in flux (selected work is shown in Table 1), that concerning vascular stem cells is not. Differentiation of vascular stem cells after injury occurs at the site of injury and in proportion to the concentration of oxygen. However, other conditions must be satisfied: lactate must be elevated and pH must be depressed. These conditions are met largely by inflammatory cells that have been stimulated by the injury, the initial hypoxia, and the elevated aerobic production of lactate (Trabold et al., 2003; Vander Heiden et al., 2009). It is likely that once the process is initiated, the effect of oxygen may be to stimulate lactate production, or at least not lower it until inflammation begins to resolve (Trabold et al., 2003; Vander Heiden et al., 2009). Continued movement and re-injury is therefore expected to enhance the angiogenic response to unstable fractures. Interestingly, the hyperplastic effect of carefully controlled traction may or may not fit this assumption.

**Table 1:** **Select works illustrating effects of oxygen on skeletal cells**.

**Measuring Oxygen at the Fracture Site**	**References**
C.T. Brighton, R.B. Heppenstall, and D.A. Labosky, An oxygen microelectrode suitable for cartilage and cancellous bone. Clin Orthop Relat Res 80 (1971) 161-6.	Brighton et al., 1971
C.T. Brighton, and A.G. Krebs, Oxygen tension of nonunion of fractured femurs in the rabbit. Surg Gynecol Obstet 135 (1972) 379-85.	Brighton and Krebs, 1972
C. Lu, M. Rollins, H. Hou, H.M. Swartz, H. Hopf, T. Miclau, and R.S. Marcucio, Tibial fracture decreases oxygen levels at the site of injury. Iowa Orthop J 28 (2008) 14-21.	Lu et al., 2008b
Lu, N. Saless, X. Wang, A. Sinha, S. Decker, G. Kazakia, H. Hou, B. Williams, H.M. Swartz, T.K. Hunt, T. Miclau, and R.S. Marcucio, The role of oxygen during fracture healing. Bone 52 (2013) 220-9.	Lu et al., 2013
**EFFECT OF OXYGEN ON FRACTURE HEALING**	
R.B. Heppenstall, C.W. Goodwin, and C.T. Brighton, Fracture healing in the presence of chronic hypoxia. J Bone Joint Surg Am 58 (1976) 1153-6.	Heppenstall et al., 1976
Lu, N. Saless, X. Wang, A. Sinha, S. Decker, G. Kazakia, H. Hou, B. Williams, H.M. Swartz, T.K. Hunt, T. Miclau, and R.S. Marcucio, The role of oxygen during fracture healing. Bone 52 (2013) 220-9.	Lu et al., 2013
**EFFECT OF OXYGEN ON STEM CELLS**	
W.L. Grayson, F. Zhao, B. Bunnell, and T. Ma, Hypoxia enhances proliferation and tissue formation of human mesenchymal stem cells. Biochem Biophys Res Commun 358 (2007) 948-53.	Grayson et al., 2007
P. Malladi, Y. Xu, M. Chiou, A.J. Giaccia, and M.T. Longaker, Effect of reduced oxygen tension on chondrogenesis and osteogenesis in adipose-derived mesenchymal cells. Am J Physiol Cell Physiol 290 (2006) C1139-46.	Malladi et al., 2006
L.F. Raheja, D.C. Genetos, A. Wong, and C.E. Yellowley, Hypoxic regulation of mesenchymal stem cell migration: the role of RhoA and HIF-1alpha. Cell Biol Int 35 (2011) 981-9.	Raheja et al., 2011
A. Wong, E. Ghassemi, and C.E. Yellowley, Nestin expression in mesenchymal stromal cells: regulation by hypoxia and osteogenesis. BMC Vet Res 10 (2014) 173.	Wong et al., 2014
C.C. Tsai, T.L. Yew, D.C. Yang, W.H. Huang, and S.C. Hung, Benefits of hypoxic culture on bone marrow multipotent stromal cells. Am J Blood Res 2 (2012) 148-59.	Tsai et al., 2012
**EFFECT OF OXYGEN ON OSTEOBLASTS**	
D. Wu, J. Malda, R. Crawford, and Y. Xiao, Effects of hyperbaric oxygen on proliferation and differentiation of osteoblasts from human alveolar bone. Connect Tissue Res 48 (2007) 206-13.	Wu et al., 2007
S.M. Warren, D.S. Steinbrech, B.J. Mehrara, P.B. Saadeh, J.A. Greenwald, J.A. Spector, P.J. Bouletreau, and M.T. Longaker, Hypoxia regulates osteoblast gene expression. J Surg Res 99 (2001) 147-55.	Warren et al., 2001
D.S. Steinbrech, B.J. Mehrara, P.B. Saadeh, J.A. Greenwald, J.A. Spector, G.K. Gittes, and M.T. Longaker, Hypoxia increases insulinlike growth factor gene expression in rat osteoblasts. Ann Plast Surg 44 (2000) 529-34; discussion 534-5.	Steinbrech et al., 2000
D.S. Steinbrech, B.J. Mehrara, P.B. Saadeh, G. Chin, M.E. Dudziak, R.P. Gerrets, G.K. Gittes, and M.T. Longaker, Hypoxia regulates VEGF expression and cellular proliferation by osteoblasts *in vitro*. Plast Reconstr Surg 104 (1999) 738-47.	Steinbrech et al., 1999
O.C. Tuncay, D. Ho, and M.K. Barker, Oxygen tension regulates osteoblast function. Am J Orthod Dentofacial Orthop 105 (1994) 457-63	Tuncay et al., 1994
**EFFECT OF OXYGEN ON CHONDROCYTES**	
E.G. Meyer, C.T. Buckley, S.D. Thorpe, and D.J. Kelly, Low oxygen tension is a more potent promoter of chondrogenic differentiation than dynamic compression. J Biomech 43 (2010) 2516-23.	Meyer et al., 2010
P. Malladi, Y. Xu, M. Chiou, A.J. Giaccia, and M.T. Longaker, Effect of reduced oxygen tension on chondrogenesis and osteogenesis in adipose-derived mesenchymal cells. Am J Physiol Cell Physiol 290 (2006) C1139-46.	Malladi et al., 2006
M. Hirao, N. Tamai, N. Tsumaki, H. Yoshikawa, and A. Myoui, Oxygen tension regulates chondrocyte differentiation and function during endochondral ossification. J Biol Chem 281 (2006) 31079-92.	Hirao et al., 2006
Y. Xu, P. Malladi, M. Chiou, E. Bekerman, A.J. Giaccia, and M.T. Longaker, *In vitro* expansion of adipose-derived adult stromal cells in hypoxia enhances early chondrogenesis. Tissue Eng 13 (2007) 2981-93.	Xu et al., 2007

To address the role of the vasculature and oxygenation during fracture healing, we performed a series of experiments to examine the relationship among mechanical stability, vascularization, oxygenation, and stem cell differentiation into chondrocytes and osteoblasts during fracture healing in mice. While tissue oxygen levels during fracture healing have been measured by other investigators in the past (e.g., Brighton and Krebs, 1972 and Table 1), the methods relied on placement of microprobes to measure oxygen levels (Brighton et al., 1971). This approach provides excellent detection methods at a single time point and location, but does not allow repeated measures at the same location. In our work, we used Electron Paramagnetic Spectroscopy (EPR) to measure oxygen levels (Khan et al., 2007). In this method, electron paramagnetic particles are implanted into the fracture site and the amount of oxygen interacting with the surface of the particle is measured via EPR. Repeated measurements can be taken on the same animal from the same location, and using histology, the position of the oxygen measurement within the fracture site can be determined.

Using this approach, we determined that oxygen levels did not differ between non-stabilized and stabilized fractures. There was an initial reduction in oxygen to hypoxic levels in both fracture groups, which rebounded within 24 h, and no differences in oxygen levels were apparent between groups by day 3. Further, we observed an increased vascular supply in the lower limb of animals with non-stabilized fractures compared to uninjured tibiae and stabilized fractures at early time points (Lu et al., 2011), which confirms results of earlier studies in large animals (Claes et al., 2002). These observations are in contrast to the prediction that mechanical instability would lead to disruptions to the vasculature supply and reduced oxygen levels, and others have observed decreased vascularity in non-stabilized fractures when observed several weeks post-injury (Lienau et al., 2005).

We also examined localized oxygen levels in order to determine the relationship between oxygen levels at early time points and tissue formation later. Using EPR, we measured tissue oxygen levels prior to and during stem cell differentiation, and then by histology determined the location of the electron paramagnetic particles to relate oxygen levels to tissue formation. Again, we observed an initial decrease in oxygen levels that rebounded quickly; however, we did not observe consistent relationships between levels of oxygen and formation of cartilage or bone. These results suggest that oxygen levels do not direct differentiation of stem cells into chondrocytes and osteoblasts that cartilage comprising the fracture callus is well perfused, which agrees with work showing that the living growth plate in mice is also well perfused (Farnum et al., 2006).

In addition to the descriptive work outlined above, we manipulated oxygen levels experimentally by creating either an ischemic, or altered oxygen environment, and assessing healing. To create an ischemic environment, we removed the femoral artery of mice and observed that healing was significantly affected. We observed delayed healing and decreased bone and cartilage formation in non-stabilized fractures, and delayed bone formation in stabilized fractures. However, we did not observe a shift in the differentiation of stem cells into chondrocytes and osteoblasts (Lu et al., 2007). Since ischemia does not address the role of oxygen in isolation, we altered systemic oxygen levels by placing animals in hypoxic and hyperoxic chambers during the healing period. Chronic hypoxia delayed healing. This has been observed previously (Heppenstall et al., 1976), and is also predicted using computer modeling approaches (OReilly et al., 2016). We did not observe a change in differentiation of osteoblasts and chondrocytes in response to altered oxygen levels. Stabilized fractures healed via intramembranous ossification, and non-stabilized fractures healed primarily by endochondral ossification, independent of the level of inspired oxygen. From these experiments, we concluded that oxygen levels were important for fracture healing generally. Indeed, others have shown that transient hypoxia may actually stimulate fracture repair (Muinos-Lopez et al., 2016).

However, in our work, oxygen was not associated with directing differentiation of osteoblasts and chondrocytes during bone healing.

Other studies have led to different conclusions. Mice that lack thrombospondin-2 (TSP2), an anti-angiogenic matricellular protein, have increased vascularity and an increase in the proportion of bone to cartilage during fracture healing. Similarly, delivery of TSP2 decreases vascularity and decreases the ratio of bone to cartilage during fracture healing (Taylor et al., 2009; Burke et al., 2013). *In silico* modeling studies have also suggested that mechanical stress combined with oxygen levels act together to regulate osteoblast and chondrocyte differentiation (Carlier et al., 2015; OReilly et al., 2016). The reasons for these discrepancies are unknown, but warrant further study. Local oxygen gradients may be more important than the absolute level of tissue oxygenation during fracture healing, as was observed for vascular repair (Knighton et al., 1981). However, assessing oxygen gradients *in vivo* is difficult.

## Oxygen can stimulate bone healing

Given the importance of the blood supply for bone healing, and the increased risk that vascular injury imposes on fracture healing, developing therapies to overcome ischemic insult may provide novel therapies to stimulate repair. One approach is to treat the ischemia directly by increasing the amount of inspired oxygen in order to increase the local tissue oxygenation levels (Lu et al., 2008b). When animals with fracture and ischemia are housed in hyperoxic chambers, healing was improved compared to normoxic controls (Lu et al., 2013). Furthermore, inspiration of 100% oxygen stimulated healing in animals with a combined lung injury and bone fracture (Kemmler et al., 2015), and hyperbaric oxygen stimulates repair of critical sized defects treated with autologous bone grafts (Grassmann et al., 2015). These observations suggest that increasing inspired oxygen in patients may aid fracture healing, but more work is required to understand safety concerns regarding the potential effect of increased reactive oxygen species (e.g., Gokturk et al., 1995).

**Figure 1:**
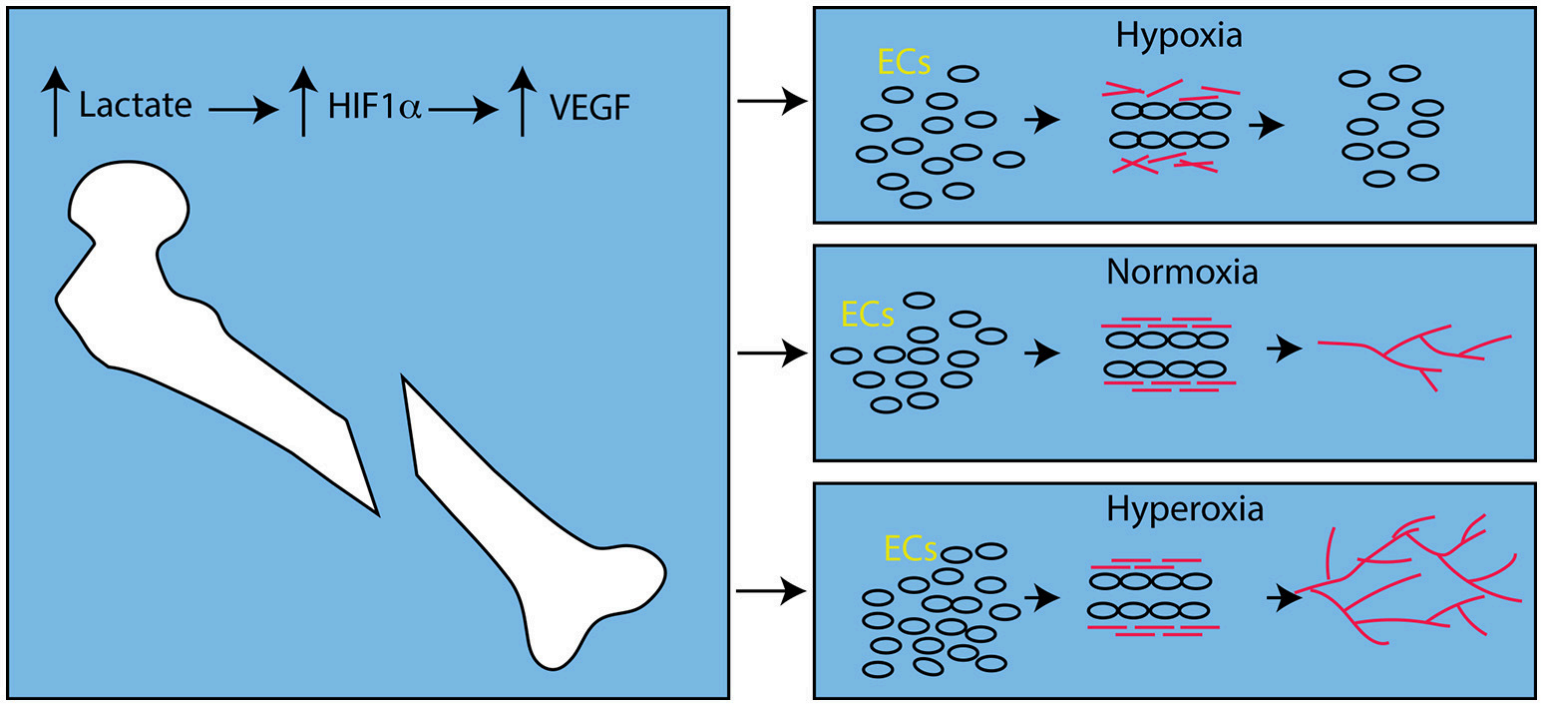
**Effect of Oxygen Levels on Angiogenesis after Fracture**. After bone fracture, lactate, produced by aerobic metabolism, stabilizes HIF1α and leads to up-regulation of VEGF. In hypoxic conditions endothelial cells (ECs, round circles) respond to VEGF, proliferate and form tubes. However, collagen (red bars) does not assemble and the vascular sprouts disintegrate. In the presence of oxygen, the collagen forms cross-links and the basement membrane stabilizes the newly formed angiogenic sprouts and angiogenesis proceeds (red lines). In hyperoxic conditions this process is amplified and angiogenesis is more robust.

Interestingly, hyperoxic animals had significantly more vasculature than animals housed in hypoxic conditions. While this agrees with previous work on the role of hyperoxia and angiogenesis (Hopf et al., 2005; Grassmann et al., 2015), in general, this is contrary to the notion that hypoxia stimulates angiogenesis by stabilizing Hypoxia inducible factor 1α (HIF1α), which increases expression of Vascular Endothelial Growth Factor (VEGF; reviewed in: Fong, 2009). Stabilization of HIF1α leads to increased expression of VEGF during fracture healing (Shen et al., 2009), but this is not necessarily due to hypoxia. Lactate inhibits activity of the prolyl hydroxylases that mark HIF1α for degradation by the proteasome, and lactate is produced by aerobic respiration in wound beds (Ghani et al., 2004; Hunt et al., 2007), including the fracture site (Lu et al., 2013). This aerobically-derived lactate is able to induce angiogenesis by induction of VEGF expression in well-oxygenated environments (Hunt et al., 2007). Hence, while hypoxia is able to induce VEGF expression and angiogenesis (Fong, 2009), the same degree of sustained hypoxia can inhibit angiogenesis (Hopf et al., 2005) due in part to failure of the basement membrane to form around new blood vessel sprouts (Hunt et al., 2007; Figure 1).

Modulating angiogenesis using biochemical signals is also a potentially valuable avenue to pursue (Carano and Filvaroff, 2003). Thrombospondins (TSP1 and TSP2) are matricellular proteins that inhibit angiogenesis. Genetic removal of TSP2 leads to increased angiogenesis after injury and rapid tissue healing (Kyriakides et al., 1999; Krady et al., 2008). During healing of ischemic fractures, removal of *TSP2* led to better healing that was accompanied by increased vascularity (Miedel et al., 2013). Similar outcomes have been observed after blocking Tsp1 activity (Isenberg et al., 2008) and blocking the TSP receptor, CD47, suggesting that this signaling pathway may be a good target for stimulating repair in patients with concomitant ischemia.

## Conclusion

Ischemia impairs fracture healing, which contributes to a significant number of complications in fracture patients, and accrues a large societal cost burden. Very few clinical studies evaluating the consequences and underlying mechanisms of prolonged hypoxia and a lack of vascularization on fracture repair have been conducted, but recent preclinical studies have isolated the effects of ischemia on stem cell differentiation and fracture healing. In contrast to opinion, oxygen levels do not appear associated with stem cell differentiation into chondrocytes and osteoblasts, and oxygen is required for robust angiogenesis during fracture healing. However, work by others shows that low oxygen is an important driver of chondrogenesis during fracture healing (Meyer et al., 2010) and may direct chondrogenesis during development (Hirao et al., 2006). These contradictory outcomes suggest that the role of oxygen in stem cell differentiation requires further study. Results from this new work may lead to novel therapies to stimulate fracture healing in patients with ischemia based on oxygen delivery (Yang et al., 2013; Koga et al., 2014) and angiogenic agents. However, further research is needed to determine the effective time-course of oxygen treatment, as well as the specific cellular processes that are affected by oxygen levels.

## Author contributions

All authors participated in the discussion and intellectual contribution that was the foundation of the article. KM, SB, and CB wrote the draft manuscript and all of the authors contributed to the revision and production of the final version. Each of the authors approved the final version to be published and agree to be accountable for all aspects of the work.

### Conflict of interest statement

The authors declare that the research was conducted in the absence of any commercial or financial relationships that could be construed as a potential conflict of interest.
